# Soil Phosphorus Dynamics are an Overlooked but Dominant Control on Mineral‐Associated Organic Matter

**DOI:** 10.1111/gcb.70307

**Published:** 2025-07-07

**Authors:** Hannah P. Lieberman, Christian von Sperber, Cynthia M. Kallenbach

**Affiliations:** ^1^ Department of Natural Resource Sciences Macdonald Campus of McGill University Sainte‐Anne‐de‐Bellevue Québec Canada; ^2^ Department of Geography McGill University Montréal Québec Canada

**Keywords:** carbon, destabilization, mineral‐associated organic matter, nitrogen, organic matter persistence, soil phosphorus

## Abstract

Understanding the controls on soil organic matter (SOM) cycling is essential for predicting how future pedoclimatic conditions will affect soil carbon (C) sequestration and emissions. However, significant uncertainty surrounds the controls on SOM formation, persistence, and destabilization, especially for the more persistent pool, mineral‐associated organic matter (MAOM). Here we argue that much of this uncertainty can be resolved by incorporating soil phosphorus (P) into conceptual and empirical models of SOM persistence because: (1) P forms the strongest bonds in the MAOM pool, resulting in greater relative enrichment and persistence; (2) this P enrichment regulates the formation of organo‐organic associations within MAOM; (3) bioavailable P drives the production of microbial necromass and by‐products, which shape contributions to MAOM; and (4) microbial P demand stimulates the destabilization of MAOM. Under this new framework, we propose specific consequences of global change, such as changes in pH and short‐term flooding, on MAOM persistence. Given soil P's outsized role in MAOM formation, a better understanding of the abiotic and biotic controls on MAOM P enables us to form more accurate predictions of MAOM persistence and destabilization under global change.

## Graphical abstract

We argue that soil phosphorus (P) plays a critical and underrated role in mineral associated organic matter (MAOM) persistence and soil organic matter cycling, as P forms strong mineral bonds, influences microbial processes, and can drive MAOM behavior under changing environmental conditions. Incorporating P into soil organic matter frameworks can help resolve uncertainties surrounding organic matter persistence under changing environmental conditions.

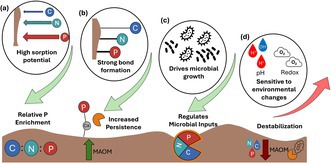

## 1 Introduction

Determining controls on soil organic matter (SOM) persistence remains central to understanding soil carbon (C) cycling and subsequent climate change feedback effects. SOM persistence originates from physical protection, sorption/desorption dynamics, and microbial processing (Balesdent 1996; Lehmann and Kleber 2015; Schmidt et al. 2011), where microbial access to and processing of SOM largely determines OM's fate in the soil (Gleixner 2013; Moinet et al. 2018). Thus, SOM is never truly persistent since it is an emergent property of the soil ecosystem and susceptible to loss with shifting biotic and environmental conditions (Kleber and Lindsley 2022). To better capture how different pedoclimatic scenarios and decomposer community dynamics influence SOM persistence, process‐based models (e.g., Waring et al. 2020; Wieder et al. 2014) have replaced previous pool‐based approaches, such as slow and fast cycling SOM models (e.g., Jenkinson 1990; Sollins et al. 2009). This shift to a process‐based approach led to an emphasis on incorporating soil nitrogen (N) as a controlling factor on the biotic and abiotic processes affecting OM retention and loss (Cotrufo et al. 2013; Daly et al. 2021; Rocci et al. 2024), and including phosphorus's (P) effects on plant productivity into terrestrial models, with implications for soil C (Goll et al. 2012; Peñuelas et al. 2013; Zhang et al. 2014). However, P is often neglected in conceptual and empirical models of belowground C persistence. We argue that soil P, though typically less abundant in soil and organisms relative to N, is likely a greater control on SOM dynamics due to its high sorption potential, stronger organo‐mineral bond formation, and unique function in microbial growth.

The C, N, and P cycles are typically coupled, where a change in one impacts the other elements, ultimately shaping the composition and behavior of SOM across a range of residence times (Celi et al. 2022). SOM with the longest residence time, based on radiocarbon and stable isotope tracing, is often associated with soil minerals (Gleixner et al. 1999; Hemingway et al. 2019; Torn et al. 1997). This mineral‐associated organic matter (MAOM) pool is the largest SOM pool, storing on average 65% of total C in mineral soils (Georgiou et al. 2022; Sokol et al. 2022). MAOM is protected through multiple concurrent mechanisms that include direct mineral associations and organo‐organic interactions, as well as biological inaccessibility within micropores and microaggregates. Both inorganic and organic P sorb strongly onto mineral surfaces, often at a higher rate and a stronger bond energy than C or N moieties, rendering it an important driver of MAOM formation and persistence (Guppy et al. 2005 and references within). Yet the focus of how soil P influences C dynamics often centers on plant and microbial P limitations and the resulting consequences for ecosystem productivity (e.g., Jindo et al. 2023; Zechmeister‐Boltenstern et al. 2015), rather than how P sorption characteristics influence MAOM formation and destabilization (but see Spohn 2020a; Spohn 2024).

**FIGURE 1:**
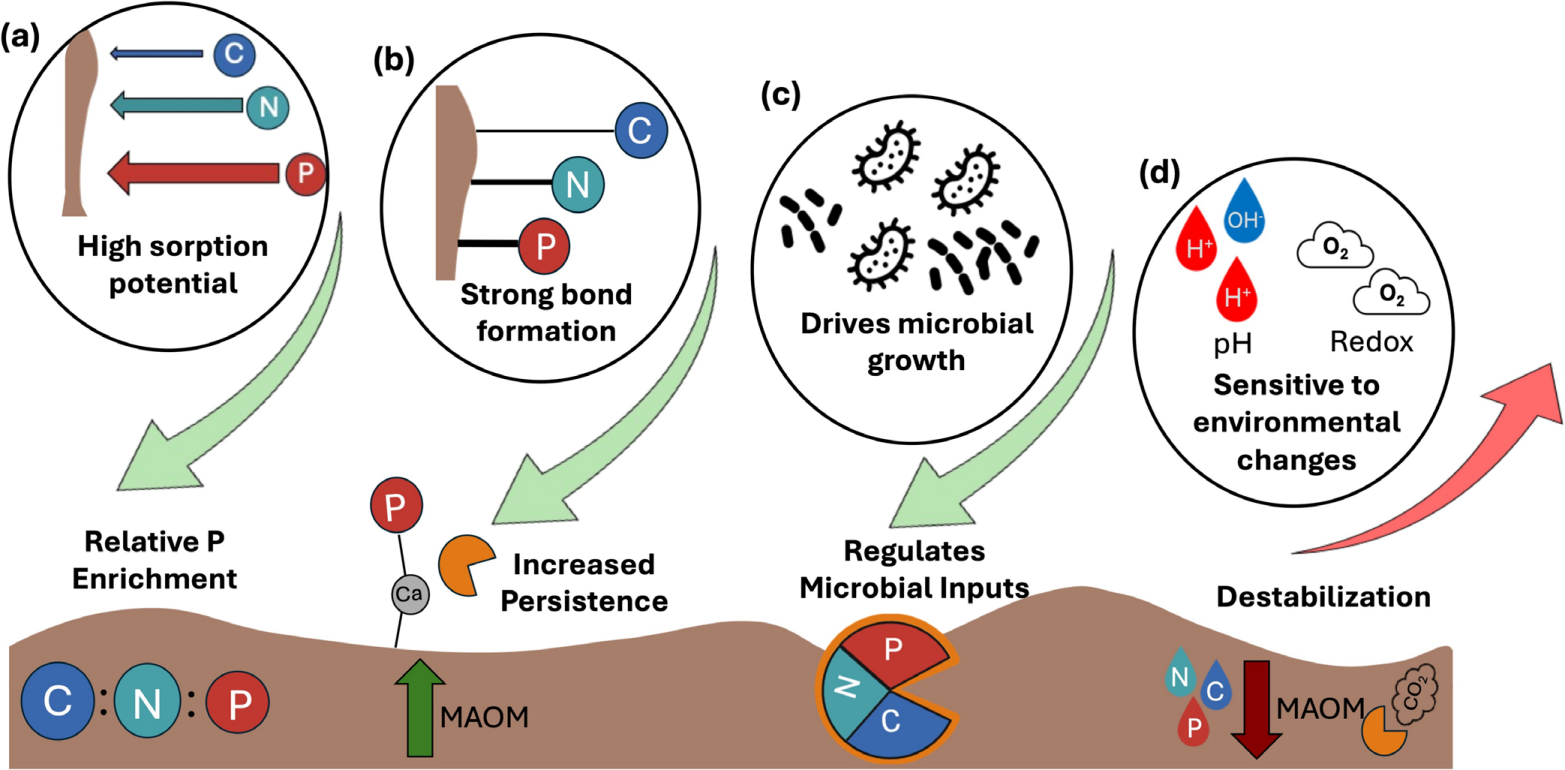
The effects of phosphorus (P) on organic matter persistence. The high sorption potential of P drives a relative P enrichment in MAOM decreasing MAOM C:N:P compared to bulk soil (a). P also typically forms stronger bonds on mineral surfaces than C or N moieties, rendering it more persistent, especially to biotic destabilization like microbial extracellular enzymes, increasing MAOM accrual and persistence (b). Soluble P drives microbial growth, necromass and by‐product production, influencing the quantity and composition of microbial inputs to MAOM (c). Yet, MAOM P is particularly sensitive to destabilization under pedoclimatic changes like shifts in pH and redox conditions, leading to loss of MAOM via leaching or respiration (d).

Here we highlight how soil P drives the accumulation and loss of slower cycling MAOM C. Inherent differences in the biogeochemistry and bio‐utilization of C, N, and P dictate SOM persistence and responses to environmental change (Celi et al. 2022). Determining these mechanistic differences, with an emphasis on P, and using them to predict the cascading consequences for SOM storage, may prove to be a powerful tool in understanding how SOM accumulates, persists, and shifts under changing environmental conditions. We first argue how P, relative to C, and N, differs in its influence on MAOM formation and persistence, and then how variances in microbial processing of C, N, and P could affect subsequent sorption/desorption MAOM dynamics. Lastly, we explore how these differences among the three elements determine when and how MAOM is vulnerable to destabilization under changing environments, using changing soil pH and flooding as examples (Figure 1).

## 2 Differences in C, N, and P Sorption Dynamics Shape MAOM Formation

MAOM consists of compounds directly bonded to mineral surfaces (organo‐mineral interactions) or attached to compounds already within MAOM (organo‐organic interactions). Several reviews discuss the relationship between MAOM and soil mineralogy, texture, and compound chemistry (Jilling et al. 2018; Kleber et al. 2021, 2015; Rasmussen et al. 2018; Spohn 2024), but MAOM accumulation and persistence are also intrinsically shaped by C, N, or P relative abundances. Here we highlight how compounds with N or P functional groups and N/P‐free compounds associate with mineral surfaces differently and compare their subsequent likelihood of accumulating and persisting as MAOM.

### 2.1 P Outcompetes C and N in the Organo‐Mineral Layer, Driving MAOM P Enrichment

The formation of organo‐mineral associations by C, N, or P functional groups depends on their sorption potential (the readiness of a compound to sorb onto a mineral and the strength of its bond). Kinetic processes of diffusion, competition, and exchange reactions further influence adsorption as they impact a compound's ability to reach a mineral surface (Kleber et al. 2021). Compounds sorb to mineral surfaces through different associations ranging from strong to weak: ligand exchange, cation bridging, anion exchange, hydrogen bonds, and van der Waals forces (Gu et al. 1994; Sokol et al. 2019). The charge and polarity of the mineral and organic matter largely determine which association will dominate during MAOM formation (Bramble et al. 2024; Rasmussen et al. 2018). Thus, the unique chemical characteristics among N, P, or N/P‐free compounds affect their ability to form and persist as MAOM (Table 1) (Mikutta et al. 2007). The sorption potential of organic N and P compounds is largely determined by their amino (NH_2_) and phosphate (${\mathrm{PO}}_{4}^{3-}$) groups, respectively. The difference in charge between these functional groups causes them to sorb onto mineral surfaces through different associations. Under typical pedoclimatic conditions, an amino group is protonated ($-{\mathrm{NH}}_{3}^{+}$) leading to a net or partial positive charge that favors electrostatic interactions directly onto negatively charged clays (Wang and Lee 1993; Yu et al. 2013). Under similar environmental conditions, phosphate groups are deprotonated with a negative charge ($H_{2}{\mathrm{PO}}_{4}^{-}$) (Spohn 2024), preferentially binding to metal oxides via ligand exchange‐surface complexation (Ognalaga et al. 1994). N/P‐free compounds and certain N‐rich compounds, like amino acids, can form strong bonds via carboxylic acid (COOH) functional groups (Gu et al. 1994), which are deprotonated in typical soil conditions (COO^−^) (Spohn 2024).

**TABLE 1:** Example of carbon (C), oxygen (O), nitrogen (N), and phosphorus (P) functional groups, the typical organo‐mineral associations they form, and examples of compounds containing each functional group that are abundant in soil organic matter.

			Organo‐mineral associations				
			pH sensitive 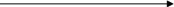				
			Weakest 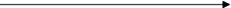 Strongest				
Functional group	Example compound	Structure	Van der Waals forces	Hydrogen bonds	Anion/Cation exchange	Cation bridging	Ligand exchange
Carbon							
Methyl (CH_3_)	n‐Hexane	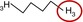	X				
Oxygen							
Hydroxyl (OH)	Glucose	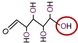	X	X			
Carboxylic acid (COOH)	Citric acid	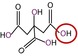	X	X	X	X	X
Nitrogen							
Amino (NH_2_)	Glucosamine	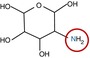	X	X	X		

Amino Acid	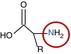					

Polypeptide	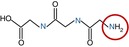					
Phosphorus							
Phosphate (PO_4_)	Phytate	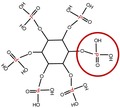	X	X	X	X	X

Myo‐inositol phosphate	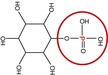					

Phospho‐glyceride	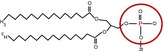					

*Note:* Although both N‐ and P‐rich compounds can have side chains with chemical characteristics that influence their sorption potential (e.g., carboxylates, aromatic rings, thiol), this table just considers their N or P functional groups.

Compounds compete for binding spaces on mineral surfaces, thought to be driven by differences in sorption potential. Inorganic P and phosphorylated organic compounds, most commonly phytate and phosphomonoesters, N‐rich compounds like amino sugars and peptides, and carboxylates, like root exudates, form organo‐mineral associations rapidly and are bound tightly, allowing them to outcompete N/P‐free or C‐rich compounds for mineral sorption sites (Guppy et al. 2005; Newcomb et al. 2017; Sollins et al. 2006). Carboxylic acids and phosphates compete more directly than aminos. Phosphates, especially monophosphates, typically display a higher sorption potential than carboxylic acids, especially in higher pH conditions and compared to mono/di carboxylates (Geelhoed et al. 1998; Hu et al. 2001; Lindegren and Persson 2010; Violante and Gianfreda 1993). This is largely because the protonated oxygens of the phosphate create an overall greater charge density, with more available OH groups on the phosphate, increasing the compound sorption potential (Celi and Barberis 2005; Magid et al. 1996).

At the whole‐soil scale, this greater sorption potential of N and P relative to C, drives N and P enrichment in MAOM compared to other SOM pools (Amorim et al. 2022; Spohn 2020a). The recent emphasis on N's role in MAOM formation is often linked to the greater propensity of N‐enriched compounds to enter and remain in the MAOM pool relative to N‐free compounds, increasing C accrual (Kopittke et al. 2018; Mikutta et al. 2010; Spohn 2024). However, we emphasize that organic P generally has a higher affinity for mineral surfaces than organic N‐rich compounds, especially with noncrystalline iron (Fe) oxides, variable charged clays, and under lower pH soils (Lin et al. 2016; Mikutta et al. 2011; Spohn 2024). When comparing MAOM to total SOM or plant litter, the increase in MAOM P:C is often greater than the increase of N:C (Spohn 2020b, 2024). For example, Adams et al. (2018) found, across varying soils, MAOM P:C was nearly six times greater than the particulate organic matter, whereas the N:C was only 1.9 times greater. While this may be partly due to preferential P microbial assimilation, the greater sorption potential of P relative to N is also likely a strong factor (Tables 1 and 2). Additions of microbial extra‐cellular polymeric substances or metabolites to soil show that P predominantly outcompetes both C and N for sorption sites, and that P has a considerably higher adsorption affinity than N (Table 2) (Cao et al. 2011; Omoike and Chorover 2006; Swenson et al. 2015). Moreover, studies comparing the sorption of a P to a N functional group within the same compound like DNA (Franchi et al. 2003; Yu et al. 2013 and sources within) and herbicides (Barja and dos Santos Afonso 2005; Jonsson et al. 2008; Sheals et al. 2002), show these compounds predominantly bind via their P group (Table 2). Thus, while our current understanding centers on N‐rich compounds creating more persistent MAOM, ample evidence suggests P‐rich compounds serve as a stronger anchor for MAOM formation and may be more important for C accrual and persistence than N in the organo‐mineral layer (Spohn 2024).

**TABLE 2:** Studies comparing sorption affinity of N and P functional groups between different compounds (extra‐cellular polymeric substances and microbial metabolites) and within the same compound (DNA and Glyphosate).

References	Relevant methods	Relevant results	Sorbents	Mineral type	Matrix	pH
**Extra‐cellular polymeric substances (EPS) and microbial metabolites**						
Cao et al. (2011)	EPS adsorption isotherms, with data fitted to the Langmuir equation.	EPS‐P compounds had an adsorption affinity (*K* L mg^−1^) 185 (montmorillonite), 8.5 (goethite), and 2.6 (kaolinite) times larger than EPS‐N	*B. subtilis* EPS	Montmorillonite, Kaolinite, and Goethite	10 mM NaCl	7.0
Mikutta et al. (2011)	EPS adsorption and co‐precipitation isotherms with varying Al(OH)_3_:EPS‐C concentrations, pH, and ionic strengths	EPS‐P showed the greatest adsorption (percentage of compounds sorbed) across treatments, and greater co‐precipitation than EPS‐N at Al(OH)3:EPS‐C higher than 0.05	*B. subtilis* EPS	Amorphous Al(OH)_3_	1.7, 17, and 170 mM NaClO_4_	3.8 and 4.5
Omoike and Chorover (2006)	EPS adsorption isotherms with varying ionic strengths, with data fitted to the Langmuir equation. Two equations were used for EPS‐P for low (P_1_) and high (P_2_) aqueous concentrations respectively	EPS‐P compounds had an adsorption affinity (*K* L m^−2^) 858 (P_1_) or 1.6 (P_2_), 696 (P_1_), and 128 (P_1_) times larger than EPS‐N in 1, 10 and100mM NaCl respectively	*B. subtilis* EPS	Goethite	1, 10, 100 mM NaCl	6.0
Swenson et al. (2015)	Bacterial lysate adsorption isotherm with varying concentrations of minerals (0.5‐32 mg in 1 mL). Assay extracts were analyzed on an LC/MS	P containing compounds showed the greatest sorption, (percentage of compounds sorbed), across treatments, with up to 100% of P containing compounds adsorbed	*P. stutzeri* RCH2 lysates	Aged Ferrihydrite	LC/MS grade water	6.8
Zhang et al. (2021)	EPS adsorption and co‐precipitation isotherms with varying Ferrihydrite:EPS concentrations	The mass fraction of adsorbed and co‐precipitated EPS‐P was greater than EPS‐N, except for C:Fe of 5 in the adsorbed system	*B. subtilis* EPS	Ferrihydrite	10 mM NaCl	4.5
**DNA/Nucleic acids**						
Beall et al. (2009)	DNA adsorption experiment followed by X‐ray diffraction to determine orientation of DNA bound to clay	Diffraction revealed that DNA binds via PO_4_^3−^ to divalent cations	Oligonucleotide Pvu4a	Montmorillonite	0.1, 1.0, 10, or 100 mM NaCl or MgCl_2_	—
Cai et al. (2007)	DNA adsorption with varying concentrations of anions: phosphate, citrate and tartrate	Anions at low concentrations for Montmorillonite and Kaolinite, and all concentrations for Goethite supressed DNA sorption, indicating that the ${\mathrm{PO}}_{4}^{3-}$ in DNA competes with anions for sorption sites	Salmon sperm DNA	Montmorillonite, Kaolinite, and Goethite	10 mM Tris Buffer	7.0
Franchi et al. (2003)	Sorption assay of nucleic acids to clays with varying concentrations of cations: Na^+^, Ca^2+^ and Mg^2+^	Adsorption of nucleic acids increased with increasing cations indicating nucleic acids bind via ${\mathrm{PO}}_{4}^{3-}$ to cations	Nucleic acids	Montmorillonite and Kaolinite	Suspension of clay	5.0–5.5
Saeki et al. (2010)	DNA adsorption experiments varying either pH, ionic strength, MgCl_2_ concentrations, or phosphate concentrations	Results revealed the primary sorption mechanisms of DNA are likely ${\mathrm{PO}}_{4}^{3-}$ binding via ligand exchange or cation bridging	Salmon sperm dsDNA	Andosol	0.1–0.5 M NaCl	3.0–9.0
**Glyphosate (herbicide)**						
Barja and dos SantosAfonso (2005)	Herbicide adsorption experiment across varying pH conditions followed by ATR‐FTIR	ATR‐FTIR results show that herbicides bind via their ${\mathrm{PO}}_{4}^{3-}$, not COOH or NH_2_, across pH treatments	N‐phospho‐methylglycine and Aminomethyl‐phosphonic acid	Goethite	0.01 M NaCl	3.0–9.2
Jonsson et al. (2008)	Herbicide adsorption experiment across varying pH conditions. Electrostatic effects evaluated with the Basic Stern model using FTIR data from Sheals et al. (2002)	Model results indicated that the compound is bound via ${\mathrm{PO}}_{4}^{3-}$ group, not COOH or NH_2_	N‐(phosphono‐methyl) glycine	Goethite	0.1 M Na(NO_3_)	3.0–10

### 2.2 P Enrichment in the Organo‐Mineral Layer Shapes Organo‐Organic Bond Formation

Organo‐organic interactions, though less stable than organo‐mineral interactions, raise the upper limit of MAOM accrual potential, sequestering higher levels of organic matter than the organo‐mineral fraction alone (Kang et al. 2024; Kleber et al. 2021). Organo‐organic interactions create three‐dimensional patchy structures that extend outwards without fully coating the mineral surface (Vogel et al. 2014). The composition of the organo‐organic layer relies on the underlying organo‐mineral layer's chemical characteristics, like compound charge or size (Chen et al. 2022; Schweizer 2022 and references within). For instance, sorbed inorganic phosphate can create a negative surface, repelling negatively charged compounds like low molecular weight organic acids that could otherwise form organo‐organic associations (Mikutta et al. 2007; Nilsson et al. 1996). Thus, sorption onto the organo‐organic layer is regulated by OM mineral surface conditioning and not mineralogy alone. Further, since the organo‐organic layer is ultimately attached to compounds in the organo‐mineral layer, OM persistence in this layer is linked to the anchor point at the mineral surface (Dippold et al. 2014). However, the relative role of N and P in surface conditioning and thus their influence on organo‐organic interactions remains unresolved (Schweizer 2022). N and P likely have an advantage over C in the more tightly held organo‐mineral layer but not necessarily in the organo‐organic layer (Kopittke et al. 2020; Zhao et al. 2020). For example, Possinger et al. (2020) found an 88% N enrichment in the organo‐mineral layer, but only 7% N enrichment in the organo‐organic layer.

**FIGURE 2:**
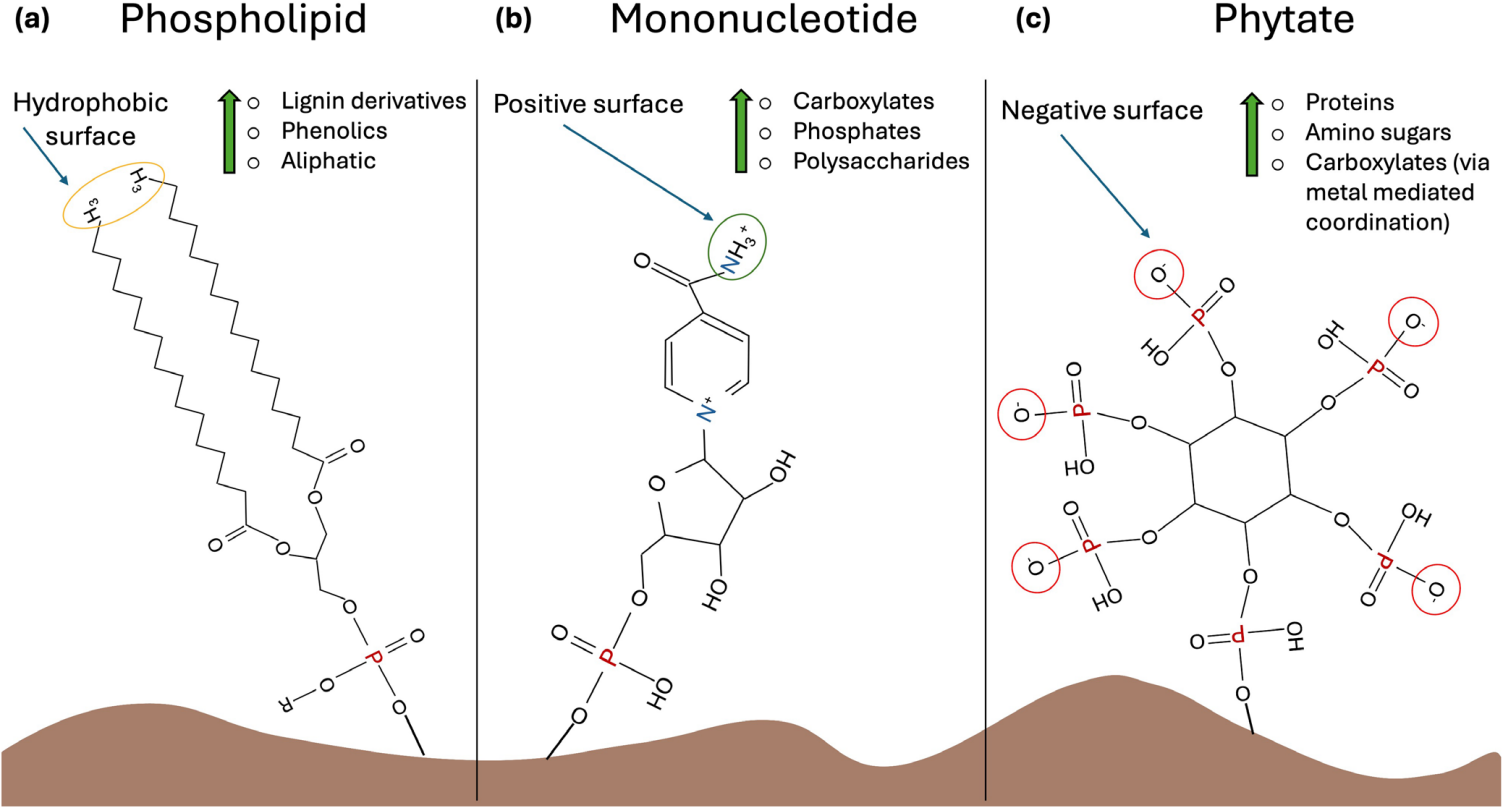
Example of how three phosphorylated compounds create new surfaces for bonding in the organo‐organic layer. Green arrows and associated compounds indicate potential preferential sorption of these compounds to the newly formed surfaces and accrual in the organo‐organic layer. When a phospholipid binds to a mineral surface, its exposed hydrophobic tail can form subsequent organo‐organic interactions, especially those with non‐polar hydrophobic functional groups (a). Similarly, a mononucleotide that binds onto a mineral surface via its phosphate group contains a positively charged amine on its tail end, creating a positively charged surface (b). In contrast, as phytate (c), contains six phosphate groups, each typically with a deprotonated oxygen, the phosphate groups that do not bind to the mineral surface create exposed negative surfaces, favoring an increase in positively charged compounds in the organo‐organic layer.

We argue that the outsized influence of P‐compounds in MAOM formation extends beyond the organo‐mineral associations to include how subsequent organo‐organic layers are formed. We suspect compounds entering the organo‐organic layer are disproportionately those most likely to bond with phosphorylated compounds due to the P enrichment and phosphorylated compounds theoretically forming stronger anchor points within the organo‐mineral layer. Phosphorylated compounds can also create more chemically heterogenous exposed surfaces available for new organo‐organic interactions, possibly inducing greater MAOM chemical diversity, a factor sometimes associated with soil C persistence (Jones et al. 2023; Lehmann et al. 2020). For example, when phospholipids, mononucleotides, or phytate sorb onto a mineral surface, they each create a new and different surface available for organo–organic interactions− hydrophobic, positive, and negative surfaces respectively (Figure 2). Phytate can make up to 50% of soil organic P, especially in aerobic and Fe‐rich soils (Darch et al. 2014; Huang et al. 2017; Kögel‐Knabner 2006 and references within). Under such conditions, we might expect that mineral surface conditioning by phytate rich organic P results in a greater negative charge, driving subsequent organo‐organic layer composition. Although still theoretical, these possible outcomes highlight the need to consider the role of P compounds in mediating organo–organic interactions as this new area of research advances.

**FIGURE 3:**
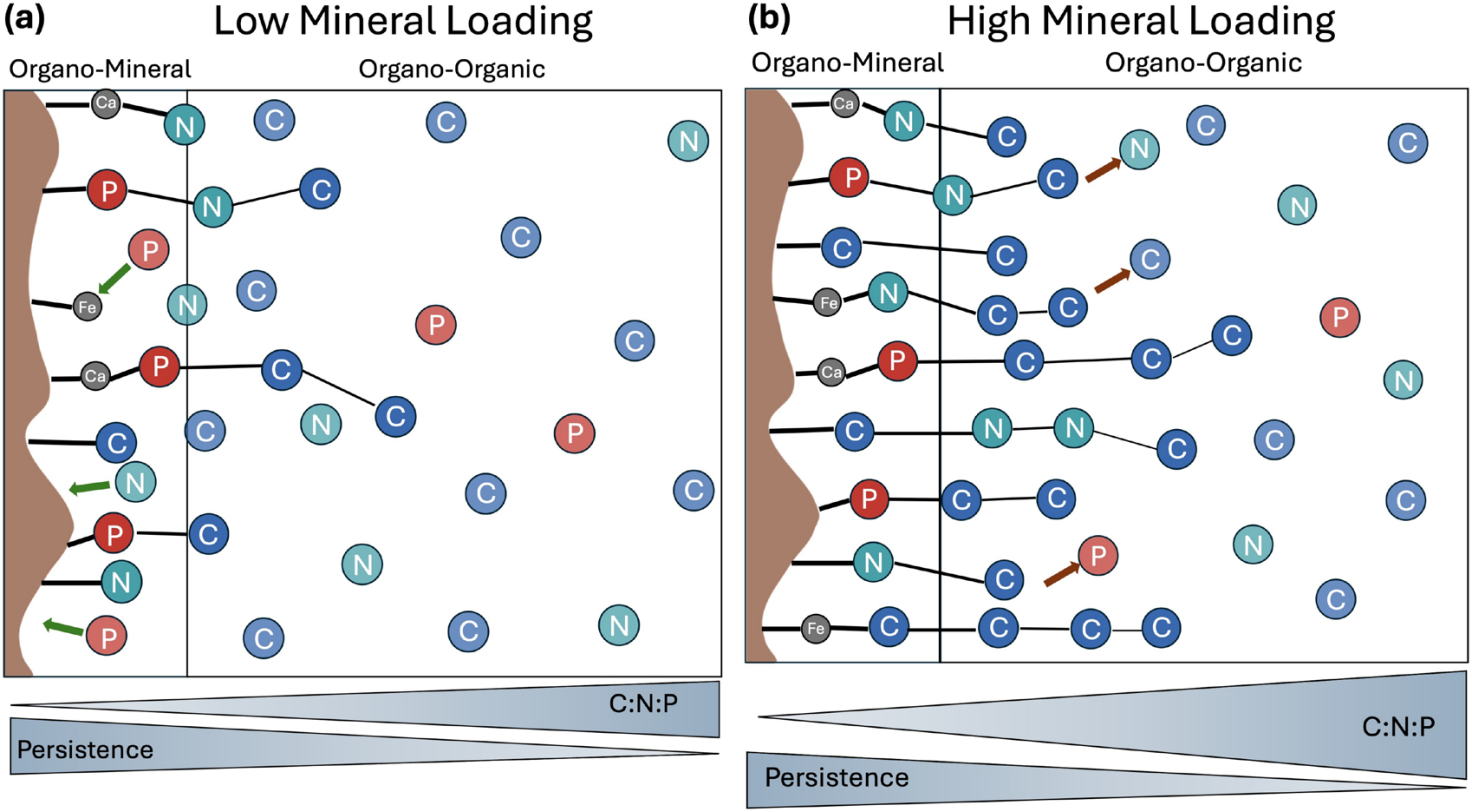
Simplified conceptual diagram of differences in the stoichiometry of the organo‐mineral and organo‐organic layers between a soil with low mineral loading (a) and high mineral loading (b). A low mineral load indicates a higher amount of MAOM sorbed in the organo‐mineral layer compared to the organo‐organic layer driving an overall lower MAOM C:N:P and increased diffusion at the mineral surface allowing for increases of sorption onto MAOM. In contrast, a soil with a highly loaded mineral surface, suggests more MAOM is stored in the organo‐organic layer compared to the organo‐mineral layer, increasing MAOM C:N:P and decreasing diffusion at the mineral surface. Soils with a highly loaded mineral surface generally have higher concentrations of MAOM compared to soils with a lower level of mineral loading. Compounds attached closer to the mineral surface are more persistent than those further out. Green arrows indicate potential sorption, an red arrows indicate potential repulsion due to electrostatic repulsion or physical blocking.

The variable controls on sorption onto the organo‐mineral and organo‐organic layer leads to distinct compositions and stoichiometries between these layers. The organo‐mineral layer typically has a lower C:N:P than the organo‐organic layer (Possinger et al. 2020; Schweizer et al. 2021). As such, we would expect that as mineral surfaces approach OM‐saturation, new MAOM associations favor organo–organic interactions with a simultaneous increase in MAOM C:N:P (Figure 3) (Kang et al. 2024). Thus, MAOM C:N:P may be a convenient way to estimate relative mineral loading that affects a soil's C sequestration potential.

## 3 Microbial Processing of P Drives MAOM Formation and Destabilization

Microbial inputs (necromass and by‐products) are a significant contributor to MAOM, contributing to 34%–50% of MAOM, and a focus of many MAOM formation studies (Angst et al. 2021; Chang et al. 2024; Whalen et al. 2022). Thus, here we reflect specifically on how microbes alter the soluble C, N, and P pools which are the primary supply of compounds available for sorption to MAOM. This soluble pool consists of inorganic ions, compounds from microbial biomass alive and dead (necromass), and highly decomposed plant material dissolved or suspended in water. Differences in the relative and absolute concentrations of soluble C, N, and P arise from plant and microbial inputs, plant and microbial C and nutrient uptake, and selective desorption (Mooshammer et al. 2014; Mori et al. 2018). These biotic influences on soluble C, N, and P content directly impact soluble OM interactions with MAOM and its subsequent retention (Chen et al. 2019; Jalali et al. 2018). Although previous theories of SOM cycling integrate C, N, and P stoichiometry within a microbial context (Cleveland and Liptzin 2007; Mooshammer et al. 2014; Zechmeister‐Boltenstern et al. 2015), this concept has not been fully expanded to the subsequent changes in the MAOM pool.

### 3.1 Microbial Growth and Biomass Allocation Depends on P Bioavailability

As microbial biomass is a major contributor to MAOM, variability in its quantity and chemical composition directly impacts the supply to MAOM (Domeignoz‐Horta et al. 2021). Microbial biomass P is distinct from biomass C and N in two significant ways that likely influence inputs to MAOM: its stoichiometric flexibility and control on microbial growth. Microbes shift their biomass C:N:P in response to changes in resources, affecting microbial C:N:P contributions to MAOM (Chen et al. 2019; Fanin et al. 2017; Scott et al. 2012). Microbial cellular components vary in their C:N:P, where a change in microbial C:N:P may occur following adjustments to elemental allocation toward growth, maintenance, or uptake (Franklin et al. 2011). Growth‐based macromolecules are particularly P‐rich, especially RNA and ATP, requiring a higher P demand compared to maintenance and uptake cellular machinery, where P is predominantly used in the phospholipid bilayer (Marklein and Houlton 2012). As P is mainly involved in the growth and less in the maintenance phase, and C and N are involved in both phases, microbes adjust their P demand more readily than C and N (Mooshammer et al. 2014; Scott et al. 2012), rendering microbial biomass C:N considerably more homeostatic than C:P (Cleveland and Liptzin 2007; Danger et al. 2008; Hall et al. 2011). As such, microbial C:P is more likely to shift with and reflect a change in environmental conditions and substrate supply.

The high P‐demand during microbial growth phases renders soluble P an important predictor of net biomass production, similar to or more so than soluble N (Chen et al. 2019; Sterner 1995). For instance, P additions increase DNA replication, but P‐limited microbes allocate C more toward maintenance and P toward phospholipids rather than growth (Chen et al. 2023; Russell 1991; Vallino et al. 1996). Changes in soluble P are also likely a greater control on microbial C cycling than changes in soluble N. Microbial carbon use efficiency and anabolic growth are more responsive with N + P or P additions than singularly N additions (Keiblinger et al. 2010; Luo et al. 2020; Zhang et al. 2023). Additionally, increases in soluble, bioavailable P are shown to increase overall rates of microbial inputs to MAOM (Luo et al. 2022). The increased inputs are likely due to both higher necromass production and a greater sorption potential of the microbial inputs with a higher P content. Accordingly, P enrichment in necromass is likely an additional explanation for MAOM accrual under high levels of microbial growth and activity. Thus, relative to microbial biomass C:N, microbial biomass C:P may serve as a better indicator of how changes in environmental conditions, especially C and nutrient inputs, shift microbial necromass and by‐product production, impacting microbial inputs to MAOM in terms of quantity and compound composition.

### 3.2 Microbial P Utilization Changes Soluble OM and MAOM Composition

Microbes both shape and respond to changes in C, N, and P bioavailability, directly impacting MAOM through nutrient mining and indirectly by altering the soluble pool composition. Microbes can destabilize MAOM through extracellular enzymes production, which depolymerizes organic compounds into more bioavailable forms to alleviate C, N, and/or P resource limitations (Jian et al. 2016). Similar or higher rates of enzymatic activities on mineral surfaces compared to particulate organic matter (Kandeler et al. 1999) and bulk soil (Brandt et al. 2023; Kandeler et al. 2019) suggest enzymes are a critical and localized MAOM desorption mechanism and partly explain how MAOM becomes biologically available (Jilling et al. 2021; Kleber et al. 2021). Enzymes that act on MAOM are largely driven by stoichiometric demand for the limiting nutrient, typically N or P (Daly et al. 2021). While there is a current focus on MAOM as an N resource, we posit that microbes use MAOM as a P resource as frequently or more often than N (Olander and Vitousek 2004; Richter et al. 2006; Turner and Joseph Wright 2014). For example, Mao et al. (2024) found that P‐acquiring enzymes were negatively correlated with MAOM C concentrations, but did not find a relationship between N‐acquiring enzymes and MAOM C. Further, typically, P‐acquiring enzymes are more responsive to changes in nutrient availability (both N and P) than N‐acquiring enzymes (Marklein and Houlton 2012; Xiao et al. 2018). As such, microbes would be more stimulated to increase P‐acquiring enzymes with an increase in soluble N:P, than they would be to produce N‐acquiring enzymes with a decrease in N:P. Therefore, all else being equal, we would expect a greater increase in enzyme destabilization and loss of MAOM in soils with a P limitation, compared to N‐limited soils.

Certain microbial specialists, and communities where their abundances are high, can especially affect MAOM sorption/desorption dynamics through P acquisition. For instance, P‐solubilizing bacteria like *Pseudomonas* spp. and *Bacillus* spp. can destabilize MAOM P through the release of mineral‐dissolving compounds like organic acid anions, siderophores, protons, and hydroxyl ions, by anion/ligand exchange and metal chelation (Etesami et al. 2021; Jones and Oburger 2011; Kaur et al. 2016). This P mining likely releases MAOM C and N ultimately attached to the phosphate group, as these compounds target the P‐mineral bond, and organo–organic bonds are dependent on the persistence of the organo‐mineral bond (Ding et al. 2021; Dippold et al. 2014). Additionally, plant root symbionts that do not use soil C for their energy needs−like N‐fixing bacteria, which have a high P demand, and arbuscular mycorrhizal fungi, which acquire and transport P to plant roots− shift the soluble pool composition as they assimilate soil P but not C, increasing C:P. We posit that in communities dominated by these symbionts, soluble C:P is elevated, increasing C diffusion toward mineral surfaces and C sorption into MAOM, regardless of its lower sorption potential, especially in systems with low mineral loading (Kleber et al. 2021). Thus, unlike generalist decomposers, these specialized microbial species that influence soluble P concentrations may have additional impacts on MAOM formation apart from their necromass contributions.

## 4 Controls on MAOM Destabilization and Consequences Under Environmental Change

MAOM is increasingly recognized as a dynamic pool, shifting with environmental conditions (Huang and Hall 2017; Jilling et al. 2020; Keiluweit et al. 2015). However, while MAOM C, N, and P are all susceptible to destabilization, the varying organo‐mineral and organo–organic bonds they form impact their likelihood to persist and their relative vulnerability to different disturbances. Currently, MAOM C and N are thought to be more susceptible to destabilization mechanisms associated with increased bio‐accessibility compared to MAOM P because of the weaker bonds that C and N typically form (Celi et al. 2022). As such, MAOM C and N are more sensitive to destabilization with environmental shifts that increase microbial access to and/or processing of MAOM, like increased pore space connectivity, increases in aerobic conditions, and release of occluded substrates (Bailey et al. 2019; Keiluweit et al. 2017). While the stronger bonds of MAOM P may be less vulnerable to biological destabilization, they are highly sensitive to pedoclimatic conditions, where changes in pH and redox largely dictate P destabilization through desorption processes (Bai et al. 2017; Lin et al. 2020).

### 4.1 Soil pH Primarily Affects MAOM P

Most soil processes are naturally acidifying, but disturbances, such as high plant biomass removal, are accelerating soil acidification (Akselsson and Belyazid 2018), while increases in saltwater intrusion and drought are leading to soil alkalinity (Sun et al. 2023). These changes in soil pH affect MAOM directly through shifts in sorption/desorption processes and indirectly by impacting microbial processes. Soil pH shapes protonation and deprotonation, and therefore the charge of compounds and mineral surfaces, impacting the formation of electrostatic associations (Table 1). MAOM persistence is generally higher at lower pH conditions (Kleber et al. 2021). Although both N and P functional groups are pH‐sensitive, phosphate protonation/deprotonation occurs at a range of pH conditions that most soils experience (pH 4–7.2), while amino groups experience changes in proton state at a very high pH rarely found in soils (Barrow 2017; Bowden et al. 1980). This means that phosphorylated compounds are more likely to be impacted by a change in pH than N‐bearing compounds. Additionally, ligand exchange with Fe/Al oxides is particularly pH‐sensitive, where low pH conditions (< 5.5), that can occur with excessive fertilization or high weathering rates, favor Fe/Al oxide bond formation (Celi et al. 2001; Gu et al. 1994). Similarly, Ca^2+^ complexation is pH‐sensitive, increasing at pH conditions > 7 (Wang and Kuzyakov 2024). As ligand exchange and Ca^2+^ complexation are primary binding mechanisms for P moieties (with their dominance varying by soil mineralogy) (Ognalaga et al. 1994), but not necessarily for C or N, MAOM P bond formation is also more pH‐sensitive regardless of the protonation state of the compound itself.

While a lower soil pH favors greater P and MAOM retention, it also coincides with reduced plant P availability (Barrow 2017; Spohn 2020a). To overcome this limitation in agricultural systems, farmers commonly apply lime (calcium or magnesium carbonates) to neutralize soil pH. Liming limits P sorption and causes MAOM P desorption, instigating a potential initial loss of P‐rich MAOM and preventing MAOM P accrual (Curtin et al. 2016). Many C‐ and lignin‐degrading, and some N‐degrading extracellular enzyme activities are optimized at a similar pH (5.5–7). However, acid and alkaline phosphatase enzymes are typically highest at lower (4.5–5.5) and higher (8.5–9.5) pH conditions, respectively (Wang and Kuzyakov 2024). Therefore, liming may simultaneously induce abiotic MAOM destabilization primarily of MAOM P and biotic destabilization of MAOM C and MAOM N via enzyme production. Yet, the increase in bioavailable P combined with a more favorable pH (5–7) following lime additions should also cause greater plant and microbial growth and biomass P concentrations (Pietri and Brookes 2008). Liming also induces a shift in microbial community composition, often favoring relatively P‐rich bacteria compared to many fungi (Lauber et al. 2008; Rousk et al. 2010). This P‐enrichment may effectively increase the sorption potential of new MAOM inputs, especially if there are further pH increases (> 7) or decreases (< 5.5). Thus, the effect of raising soil pH on total MAOM stocks likely depends in part on whether new MAOM inputs counteract losses of existing MAOM associated with a/biotic destabilization.

### 4.2 MAOM C:N:P Determines Destabilization Under Flooding

**FIGURE 4:**
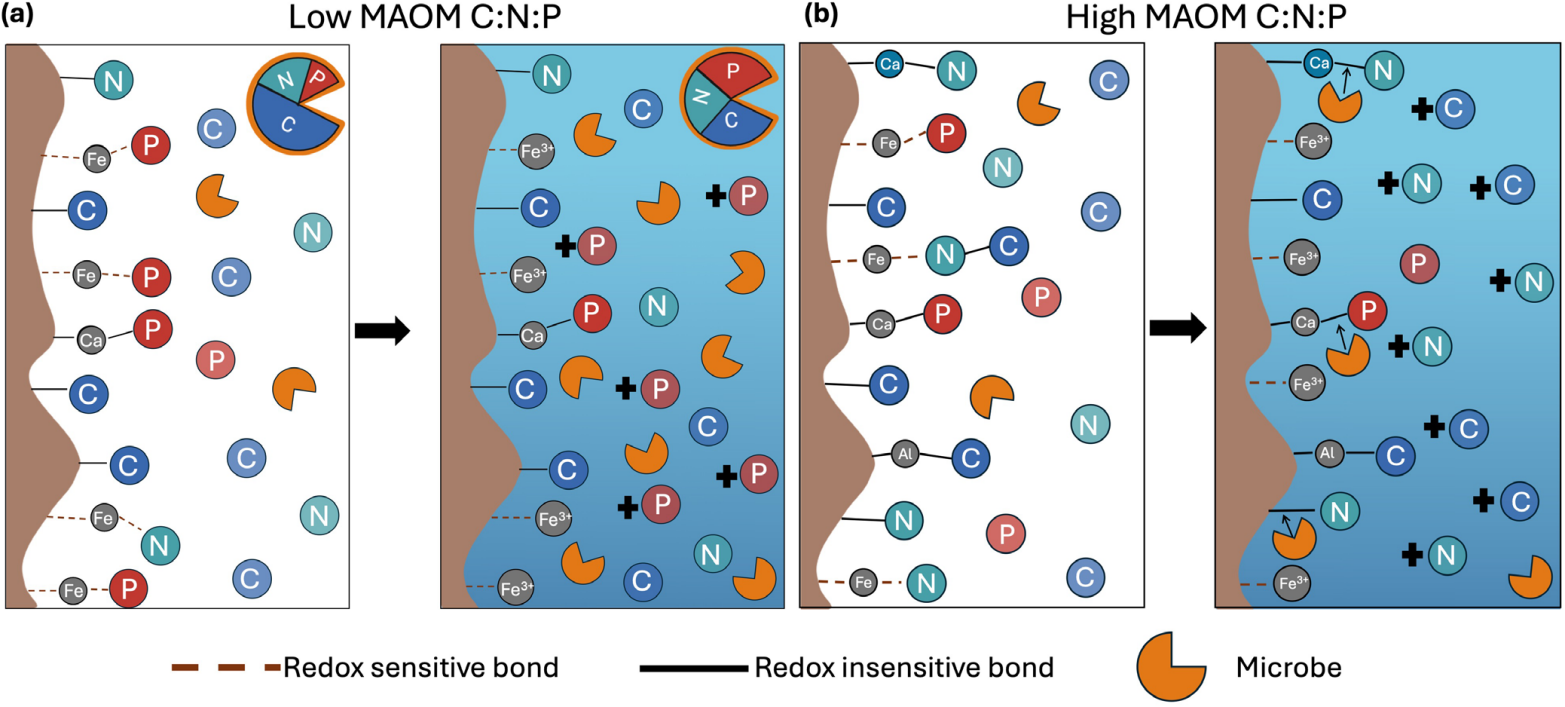
Differences in microbial responses to flooding based on initial MAOM stoichiometry. With a low MAOM C:N:P, MAOM destabilization releases relatively high levels of soluble and bioavailable P. This increase stimulates P uptake, decreasing biomass C:N:P and increasing microbial growth (a). With a high MAOM C:N:P, relatively lower levels of P and higher levels of C and N desorb with flooding. The P limitation can stimulate nutrient mining and MAOM destabilization (b).

With climate change, shifts in pedoclimatic conditions will have multiple concurrent consequences for MAOM formation and destabilization. Here we use the example of flooding to illustrate how global change will directly and indirectly impact the controls on MAOM described thus far and how we can use our understanding of P cycling to more accurately predict consequences for MAOM. Periodic soil flooding is increasing globally in frequency and duration (Hirabayashi et al. 2021). During flooding when soils become water‐saturated, reduction–oxidation (redox) conditions, a major control on organo‐mineral bonds, are altered. Low redox conditions that dominate in anoxic, water‐saturated soils, result in the reductive dissolution of Fe and manganese (Mn), causing mineral associated C, N, and P to desorb into their respective soluble pool (Gross et al. 2020; Lin et al. 2018). However, the relative sensitivity of MAOM to flooding should depend on the MAOM C:N:P. MAOM with a lower C:P, containing high levels of inorganic P and phosphorylated organic compounds bound to Fe, will be more susceptible to destabilization under flooding than MAOM with a higher C:P (Huang et al. 2017). MAOM with a higher C:P indicates that more MAOM consists of C‐rich compounds bound by redox‐insensitive interactions, like hydrophobic bonds (Voet et al. 2013). Thus, we might expect that the relative losses from MAOM during a flood event is based on initial MAOM stoichiometry, where a lower C:P results in more MAOM lost with flooding (Figure 4).

Yet this MAOM loss may be mitigated or exacerbated by the microbial community's flood response. As flooding induces MAOM desorption, it subsequently increases soluble, bioavailable C, N, and P, which could induce shifts in microbial activity during and post‐flood. This increase might explain why heterotrophic microbial activity is not necessarily hindered and can even increase with flooding despite oxygen limitations (Patel et al. 2021; Steinweg et al. 2012). As flooding should favor P desorption, the subsequent decrease in bioavailable C:P may stimulate microbial growth leading to future increases in microbial inputs to MAOM. In contrast, an increase in soluble C:P could occur with soil disaggregation during the early flood period, increasing enzyme activity, causing further MAOM destabilization.

We can expand this understanding to hypothesize how different ecosystems respond to flooding, as MAOM destabilization mechanisms change with mineralogy, initial pedoclimatic conditions, and microbial communities (Bramble et al. 2024; Rasmussen et al. 2018). For example, both tropical forest soils and permafrost (or high‐latitude) soils typically have high concentrations of P bound to MAOM, especially to Fe and aluminum (Al), resulting in low levels of soluble P (Gross et al. 2020; Weintraub 2011 and references within). Yet, they differ in their microbial activity and limiting nutrient. In tropical soils, microbes are typically P‐limited and have relatively high metabolic activity (Camenzind et al. 2018). In permafrost soils, however, microbial communities are more often slow‐growing and N‐limited (Sistla et al. 2012). Thus, while we might expect a similar abiotic response to flooding in both ecosystems, where flooding causes a large desorption of MAOM P, the microbial responses likely diverge. In the tropics the higher microbial activity and demand for P would drive rapid uptake of the newly released P, increasing necromass and by‐product compounds available for resorption post flood, and simultaneously preventing ecosystem P loss. In contrast, in permafrost, microbial uptake will still be limited by low temperatures and N availability, leading to lower P uptake and new biomass production, leaving the newly released P vulnerable to loss via leaching. Moreover, the increase in soluble OM from desorption combined with permafrost N‐limitations may induce increases in enzyme activity to relieve N limitations, prompting additional destabilization of MAOM.

## 5 Outlook

We argue that soil P plays a critical and outsized role in MAOM persistence and SOM cycling, with the behavior of P often driving MAOM persistence. P also regulates microbial growth and OM utilization, rendering microbial biomass and soluble C:P an underrated indicator of MAOM formation and destabilization. Given MAOM P's unique properties, the relative abundance of P in MAOM likely determines whether and to what degree MAOM is vulnerable to loss under global changes like pH shifts and flooding. We propose that incorporating MAOM P into empirical and theoretical C models will help resolve many of the current challenges in predicting global C cycling, especially as related to MAOM formation, persistence, and destabilization.

Current terrestrial carbon models already incorporating P have reduced uncertainty and shifted C cycling predictions (e.g., land surface) (Goll et al. 2012; Peñuelas et al. 2013; Zhang et al. 2014), yet rely on total soil P concentrations or generalized pool turnover values, constraining model accuracy (Helfenstein et al. 2024). However, empirical data that couple MAOM P and C dynamics are severely limited (Reed et al. 2015; Wieder et al. 2015). For instance, we found only 12 studies measuring both MAOM C and P in the past decade (Table S1). Further, there is little methodological consistency in quantifying MAOM C, N, and P*—*MAOM C and N are fractionated by size or density, whereas MAOM P is often isolated through chemical extractions—rendering direct comparisons difficult. Thus, including belowground soil P processes into predictive and quantitative models first requires that more studies measure MAOM C, N, and P with consistent methods across diverse ecosystems and soil conditions. We recognize that our proposed hypotheses do not consider all possible exceptions and nuances across variable soil conditions, further highlighting the need for developing larger MAOM C, N, and P open access datasets to better identify where and when soil P is a dominant control on MAOM persistence. Based on the above sections, we propose the following areas of emphasis for building such datasets:

1. Determine if there is a consistent MAOM C:N:P threshold across soils and environmental conditions that can be used as an indicator for when less persistent, organo‐organic associations are favored.
2. Investigate how soluble C:N:P impacts microbial by‐product and necromass chemistry and in turn MAOM composition.
3. To better predict MAOM C losses, identify pedoclimatic conditions, regions, and land uses where MAOM P will be most sensitive to destabilization under climate change.

## Author Contributions

**Hannah P. Lieberman:** conceptualization, visualization, writing – original draft, writing – review and editing. **Christian von Sperber:** supervision, writing – review and editing. **Cynthia M. Kallenbach:** conceptualization, resources, supervision, writing – review and editing.

## Conflicts of Interest

The authors declare no conflicts of interest.

## Supporting information


Table S1

## Data Availability

The authors have nothing to report.
