# Supplementary material for: Soil Phosphorus Dynamics are an Overlooked but Dominant Control on Mineral‐Associated Organic Matter
Source: Glob Chang Biol. 2025 Jul 7;31(7):e70307. doi: 10.1111/gcb.70307 (PMC12230779; doi:10.1111/gcb.70307)
Supplement: Supplementary file 1 — Table S1 [file GCB-31-e70307-s001.pdf]

Soil phosphorus dynamics are an overlooked but dominant control on mineral-associated organic matter

Authors and affiliations

Hannah P Lieberman*[1], Christian von Sperber[2], and Cynthia M Kallenbach[1]

[1] Department of Natural Resource Sciences, Macdonald Campus of McGill University, Sainte-Anne-de-Bellevue, Québec, H9X 3V9, Canada

[2] Department of Geography, McGill University, Montréal, Québec, H3A 0B9, Canada

* Author for correspondence: Hannah Lieberman; hannah.lieberman@mail.mcgill.ca

Supplemental Information

Table S1. Experimental studies published in the last decade (Jan. 1, 2015-April 24, 2025) that measured the mineral fraction of phosphorus (P) and carbon (C). Studies included were found on Web of Science using the search terms “soil phosphorus” and “soil carbon” with either “particle size fraction,” “mineral associated organic matter,” “density fractionation,” or “Hedley fractionation”. Studies were only included if they measured P in either size (<63 µm), density (heavy fraction) or chemical (i.e. NaOH extracted, HCl extracted, residual or occluded) fractions *and* C in size or density fractions.

Reference	Ecosystem and Location	Depth (cm)	P Fractionation Method	C Fractionation Method
Adams et al. (2018)	Semi-natural and pasture; River catchments across the United Kingdom	0-27	Density fractionation using sodium polytungstate solution (specific gravity 1.6 g cm^{-3}).	Same as P
Cabreira et al. (2022)	Eucalyptus plantation; State of Rio de Janeiro, Brazil	0-10	Sequential chemical fractionation according to (Bowman & Cole, 1978)	Dispersion with $NaHMPO_4$ followed by size fractionation (<53 µm)
Hoosbeek et al. (2023)	Tropical rainforest; Amazonas, Brazil	0-20	Density fractionation using sodium polytungstate solution (specific gravity 1.6 g cm^{-3}).	Same as P
Khan et al. (2023)	Cropland, Shaanxi Province, China	0-20	Aggregate size fractionation (<63 µm), followed by a Hedley fractionation on aggregate size class	Aggregate size fractionation (<63 µm)
Lei et al. (2025)	Forest floodplain; Falkensteiner Vorwald region, Germany	0-100	Sonication followed by size fractionation (<20 µm).	Same as P
Lieberman et al. (2023)	Cropland; Quebec, Canada	0-15	Modified Hedley fractionation.	Dispersion with $NaHMPO_4$ followed by size fractionation (<53 µm).
Pang et al. (2024)	Alpine grasslands; Tibetan alpine grasslands, China	0-50	Sonication followed by size fractionation (<2 µm for clay; 2-53 µm for silt).	Same as P
Stahr et al. (2018)	Acid forest stand, Across Germany	0-13; 73-106	Density fractionation using sodium polytungstate solution (specific gravity 1.6 g cm^{-3}) combined with sonication	Same as P

Sugihara et al. (2017)	New and old forest, South Cameroon Plateau, Cameroon	0-100	Dispersion with NaCl followed by size fractionation (<53 µm)	Same as P
Von Sperber et al. (2017)	Semi-arid grassland; Free State Province, South Africa	0-20	Sonication followed by size fractionation (<2 µm for clay; 2-20 µm for silt) and a modified Hedley fractionation on the size fractions.	Sonication followed by size fractionation (<2 µm for clay; 2-20 µm for silt)
Xu et al. (2025)	Cut slope; Sichuan province, China	0-10	Size fraction to <53 µm with dry sieving.	Same as P
Zhang et al. (2024)	Cropland; Gongzhuling City, China	0-20	Modified Hedley Fractionation	Shaken in DI water followed by wet sieving to <53 µm

33 References

Adams, J. L., Tipping, E., Thacker, S. A., & Quinton, J. N. (2018). An investigation of the distribution of phosphorus between free and mineral associated soil organic matter, using density fractionation. *Plant and Soil*, *427*, 139-148.

Bowman, R., & Cole, C. (1978). An exploratory method for fractionation of organic phosphorus from grassland soils. *Soil Science*, *125*(2), 95-101.

Cabreira, W. V., Pereira, M. G., de Carvalho Balieiro, F., Chaer, G., dos Santos, R. N., dos Santos, F. M., & da Rocha, P. V. (2022). Soil organic phosphorus (P) increases in mixed plantations of Acacia mangium and Eucalyptus urograndis in Planosol from Brazil. *Trees*, *36*(3), 1017-1027.

Hoosbeek, M. R., Schaap, K. J., & Quesada, C. A. (2023). Carbon, nitrogen and phosphorous contents, related enzyme activities and organic matter fractions of litter and soil in a terra firme forest in Central Amazonia. *European Journal of Forest Research*, *142*(5), 1069-1079.

Khan, A., Guo, S., Wang, R., Zhang, S., Yang, X., He, B., & Li, T. (2023). An assessment of various pools of organic phosphorus distributed in soil aggregates as affected by long-term P fertilization regimes. *Soil Use and Management*, *39*(2), 833-848.

Lei, K., Bucka, F. B., Just, C., van Grinsven, S., Floßmann, S., Dannenmann, M., Völkel, J., & Kögel-Knabner, I. (2025). Distinct impact of land use and soil development processes on coupled biogeochemical cycling of C, N and P in a temperate hillslope-flood plain system. *Biogeochemistry*, *168*(1), 21.

Lieberman, H. P., Rothman, M., von Sperber, C., & Kallenbach, C. M. (2023). Experimental flooding shifts carbon, nitrogen, and phosphorus pool distribution and microbial activity. *Biogeochemistry*, *165*(1), 75-90.

Pang, B., Ma, X., Hong, J., Du, Z., & Wang, X. (2024). Effect of aridity and soil texture the profile pattern of soil C: N: P stoichiometry in Tibetan alpine grasslands. *Catena*, *245*, 108277.

Stahr, S., Graf-Rosenfellner, M., Klysubun, W., Mikutta, R., Prietzel, J., & Lang, F. (2018). Phosphorus speciation and C: N: P stoichiometry of functional organic matter fractions in temperate forest soils. *Plant and Soil*, *427*, 53-69.

Sugihara, S., Shibata, M., Mvondo-Ze, A. D., Araki, S., Kosaki, T., & Funakawa, S. (2017). Soil phosphorus of stable fraction differentially associate with carbon in the tropical forest and savanna of eastern Cameroon. *Soil Science and Plant Nutrition*, *63*(6), 616-627.

Von Sperber, C., Stallforth, R., Du Preez, C., & Amelung, W. (2017). Changes in soil phosphorus pools during prolonged arable cropping in semiarid grasslands. *European Journal of Soil Science*, *68*(4), 462-471.

Xu, Z., Sheng, M., Ai, S., Yang, Q., Li, Z., Chen, T., Kou, J., Ai, Y., & Ai, X. (2025). Soil Aggregate Stability and Characteristics of Nitrogen, Phosphorus, and Potassium Contents in Cut Slope Soils With Different Aspects in Plateau Areas. *Land Degradation & Development*.

Zhang, N., Wang, Q., Chen, Y., Zhang, S., Zhang, X., Feng, G., Gao, H., Peng, C., & Zhu, P. (2024). Phosphorus Distribution within Aggregates in Long-Term Fertilized Black Soil: Regulatory Mechanisms of Soil Organic Matter and pH as Key Impact Factors. *Agronomy*, *14*(5), 936.
